# Supplementary material for: Unveiling coherently driven hyperpolarization dynamics in signal amplification by reversible exchange
Source: Nat Commun. 2019 Jan 23;10:395. doi: 10.1038/s41467-019-08298-8 (PMC6344499; doi:10.1038/s41467-019-08298-8)
Supplement: Supplementary file 1 — Supplementary Information [file 41467_2019_8298_MOESM1_ESM.pdf]

Supplementary Information for:

Unveiling Coherently-Driven Hyperpolarization Dynamics in Signal Amplification By Reversible Exchange

Warren et. al.

Supplementary Note 1: Quantum Monte Carlo Simulations

To simulate the SABRE system, a Markov Chain Monte Carlo algorithm for chemical exchange was used to sample the PTC lifetimes. It is important to note that propagation was performed by propagating the initial density of 60%$S_{0,H}$/40%$T_{0,H}$ density forward 1 ms for each step of the simulation. First, a list of 150 randomly generated numbers (for a 150 ms simulation) between 0 and 1 and are called the set $\mathcal{S}$. If the nth element of $\mathcal{S}$, $\mathcal{S}_\mathrm{n} < \frac{k_\mathrm{d}}{1000}$, then this $\mathcal{S}_\mathrm{n}$ would define a dissociation event for the catalyst and the polarization at this time would be accumulated. Otherwise, if $\mathcal{S}_\mathrm{n} > \frac{k_\mathrm{d}}{1000}$, then the polarization was saved as $P(t)$. This process is repeated for the time-course of the simulation. To include the effect of a polarization inactive state, the polarization recorded at each of the break points is extended for a length of time T_inactive, which simulates no evolution of the PTC for that period, which is valid only at high field. These time courses were then calculated over 1600 iterations and averaged to produce the result.

The code to perform this simulation was written as part of the Warren lab's in-house spin dynamics simulation package, RogueSpin. A copy of all the necessary functions to perform the Monte Carlo calculations is provided at the end of this document.

Supplementary Note 2: Coherent SHEATH Optimization

Details about determination of relevant NMR parameters (*J*-coupling, linewidth), magnetic evolution field optimization, temperature dependence, as well as optimization of hydrogen flow rate and sample composition have been published elsewhere (See reference [1] and its supplementary material).

The sample is placed inside the magnetic shield with a small current applied to a solenoid inside the shield (See Supplementary Figure 1). A well-defined flow of *para*-hydrogen, typically 200 sccm/min, is bubbled through the sample at a given field until polarization is saturated (90 s). The sample is then transferred the high field magnet for detection. The reference experiment is repeated in regular intervals to check reproducibility and validate pulsed SABRE results.

Note that polarization buildup resembles a T_1-process. Furthermore, polarization is directly proportional to the flow rate of *para*-hydrogen. Spectra are acquired on a Bruker DX 360 (8.45 T) magnet and processed with MNova. Polarization levels are determined by comparison to a reference of neat ^{15}N-CH_3CN (19.4 M, 99.8% enrichment, CIL).

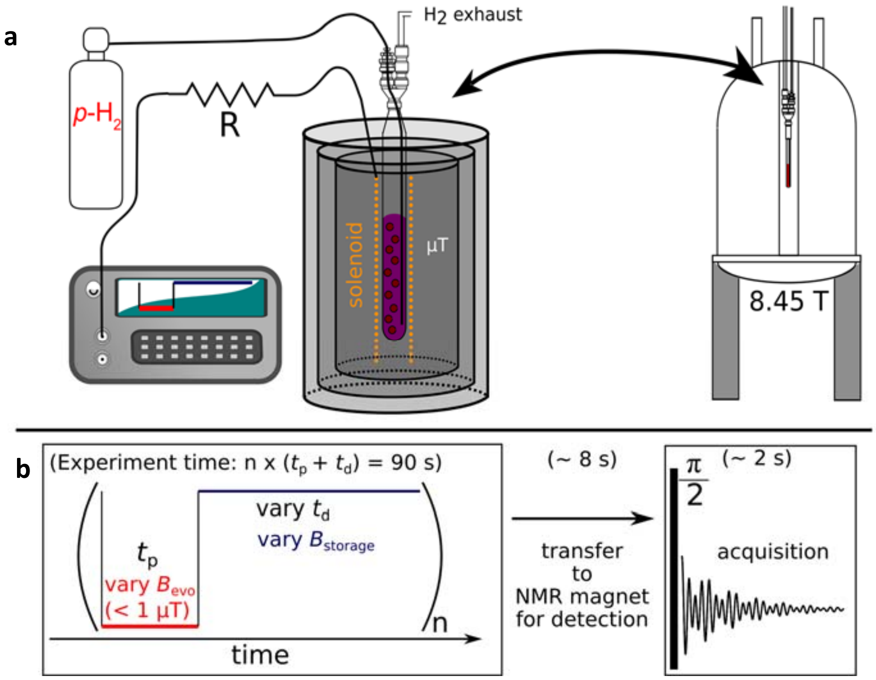

***Supplementary Figure 1 | Setup for Coherent SABRE experiment*. a** Schematic representation of the experimental setup. Shown are para-hydrogen supply, voltage source/oscilloscope and resistor to supply constant or time dependent current to the solenoid inside the three-layer µ-metal shield, the NMR flow-tube, and 8.45 T magnet for detection. **b** Procedure of the pulsed evolution field method. After the pulse sequence consisting of n repetitions of a block of evolution period at field B_{evo} and duration t_p followed by a storage period with duration t_d and field $B_{storage}$, the sample is transferred to the high-field magnet for detection.

In the pulsed field experiments, a sample is bubbled with *para*-H_2 at the same flow rate as in the reference experiments. A pumping field is applied for a duration t_p, followed by a storage field period with duration t_d (see Figure 5 in main text). To that end, a small voltage is generated by a function generator and dropped off by means of a series resistor to obtain the desired

magnetic field. The evolution storage-field procedure is repeated n times until the overall experiment time matches the reference dc-field experiment (90 s).

To investigate the dynamics of the SABRE SHEATH mechanism, we performed experiments with two different sample compositions, different magnetic field strengths B_{evo} and B_{storage} and varied the durations of the evolution period t_{p} and storage period t_{d}.

In first experiments, we attempted to vary the magnetic field during t_p and t_d, such that $B_{\text{storage}} << B_{\text{evo}}$. The storage field is then defined by the shielding factor of the µ-metal shield, and too small to determine experimentally (without nontrivial effort to detect sub-microtesla dc-fields). This approach yielded poor results, showing no improvement over dc-field SABRE-SHEATH (results not shown). This is because the dynamics near zero field are highly complex. Instead, the storage field was thus chosen as the largest field we are comfortable generating inside the shield for extended time (50 µT), as this will be significantly off resonance from the matching condition.

Variation of the magnetic pumping field B_{evo} yielded interesting results and allowed to probe internal dynamics of the spin system in an interesting way. In Supplementary Figure 2, we show a comparison between two experiments using different values of B_{evo} at constant $B_{storage}$. Overall experimental time and length of an experiment block ($t_{\text{p}} + t_{\text{d}}$), as well as number of blocks, n, are constant.

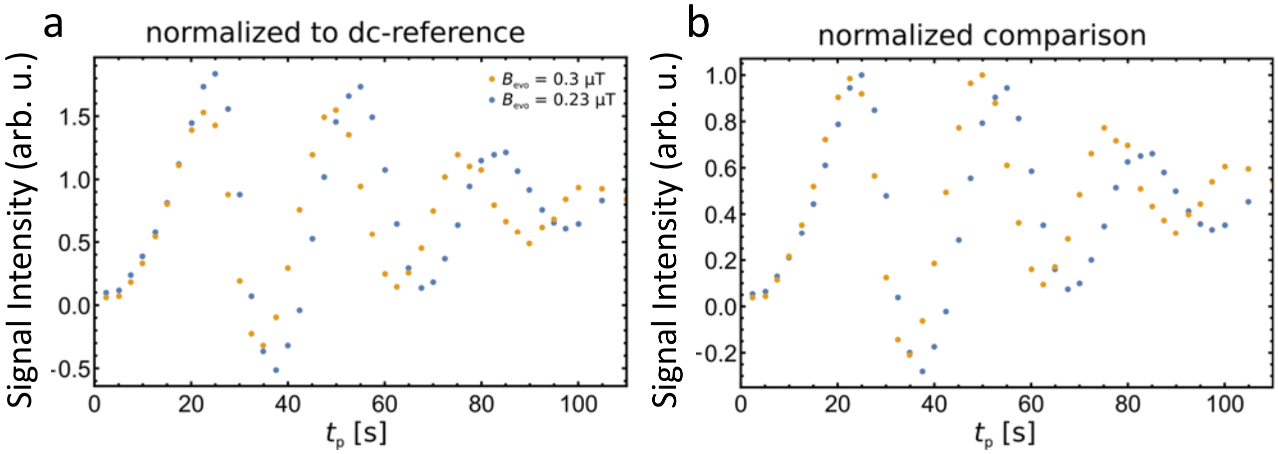

Supplementary Figure 2 | Pulsed SHEATH experiments. **a** Comparison of signal intensity as a function of t_p at two different values of B_{evo} normalized to a dc reference at the respective low and high B_{evo}. At the optimal evolution field, a higher enhancement over dc signal is obtained. For comparison of the oscillation frequency, all data has been normalized to unity **b**. As expected from theory, higher field gives rise to higher oscillation frequency.

Supplementary Note 3: Coherent DARTH Optimization

Scanning the B1 power for fully ramped (down to 0 Hz) DARTH pulses provides a maximum enhancement at ~ 15 Hz (Supplementary Figure 3) for 350 ms pulses.

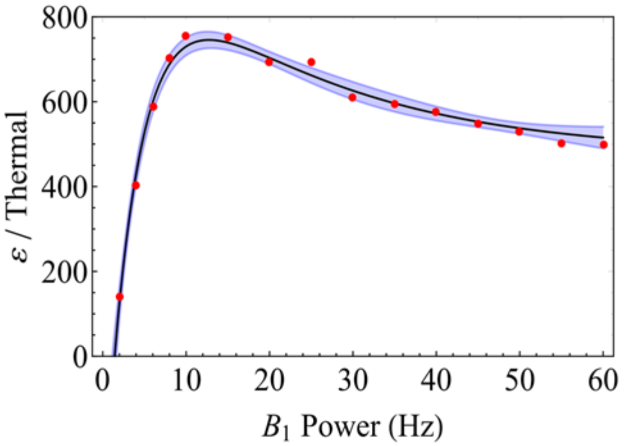

Supplementary Figure 3 | Enhancement over thermal as a function of the initial B_1 power. This is a measure of the exchange-dominated polarization, which is maximum at ~13 Hz B_1.

The pulse length and delay length in the domain dominated by incoherent dynamics were optimized (Supplementary Figure 4) such that the experiment length was a total of 60 s (approximately the 2T_1 of the nitrogen of pyridine).

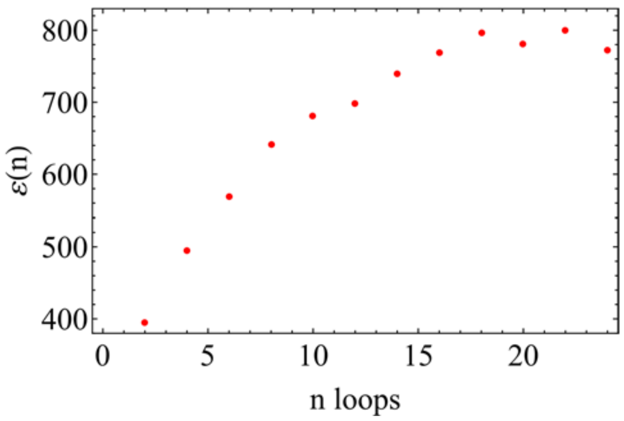

Supplementary Figure 4 | Enhancement over thermal as a function of the number of loops. Polarization is maximized and limited by the relaxation of the ^{15}N.

Having optimized the initial B_1 power at 15 Hz, the optimized ε for the core component of DARTH-SABRE is $\varepsilon = 700$ at $\Omega = -20$ Hz. However, this may be further improved by pulsing on the hydrides in-between the ramped pulses. This has the effect of refocusing the hydrides into states that may be used for further hyperpolarization or that prevent polarization annihilation. The optimized DARTH-SABRE pulse sequence is shown in Figure 1 of main text. Using an initial B_1 power of 15 Hz, $\tau_p = 800$ ms, and $\tau_d = 600$ ms, an additional factor of ~2 enhancement is achieved by refocusing the hydrides.

For DARTH-SABRE, the φ-pulse acts to invert the T_H^{-1} population established on the hydrides after the first DARTH-SABRE pulse. For experiments performed at 8.45 T, φ was optimized to be 90°.

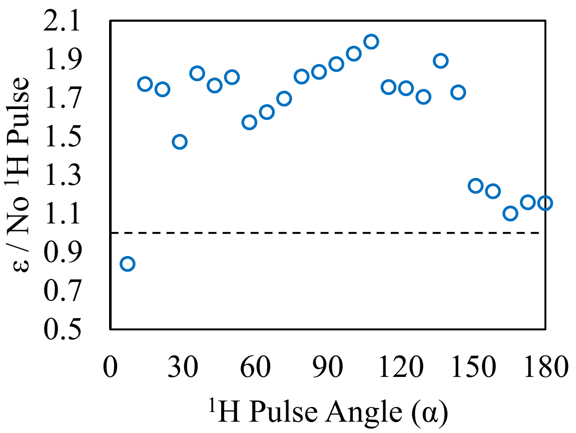

Supplementary Figure 5 | Effect of refocusing on the DARTH experiment. Enhancement over no refocusing pulse as a function of the refocusing pulse angle.

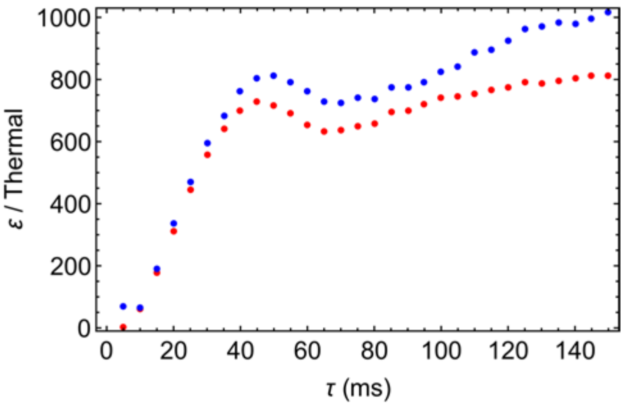

Supplementary Figure 6 | Enhancement as a function of the pulse length an initial B_1 power of 15 Hz. The red trace is at a pulse gradient of 21 Hz/s and the blue trace is at a pulse gradient of 42 Hz/s.

Corresponding to the discussion in the text referring to the oscillation frequency of the dynamics, the scan of the coherent dynamics at an initial B_1 power of 15 Hz is shown in

Supplementary Figure 6. Clearly, the oscillations are significantly damped by the exchange dynamics by time that the enhancement first reaches a maximum. For this reason, the initial B_1 power was raised to 21 Hz (data shown in paper), so that the oscillation frequency was faster and (initially) did not incur significant damping by the exchange dynamics.

^{14}N-NMR was performed on the natural abundance DARTH complex to interrogate the lifetime of the quadrupolar nucleus in the iridium-bound state (Supplementary Figure 7). The linewidth (in the extreme narrowing limit) gives $5T_Q$ = 11 ms and exhibits significantly more narrow resonances than the neat ^{14}N pyr.

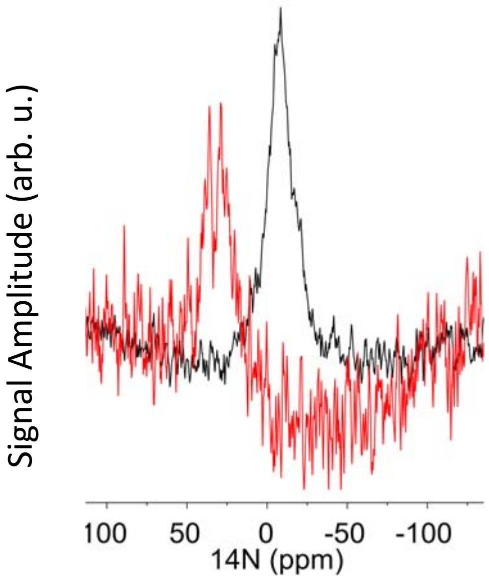

Supplementary Figure 7 | 14N spectra of SABRE sample. The black spectrum shows the resonance of free ^{14}N-pyridine and the red-spectrum shows the narrowed resonance of the bound ^{14}N-pyridine iridium dihydride complex. Both spectra were taken in methanol.

1 Colell, J. F. *et al.* Generalizing, Extending, and Maximizing Nitrogen-15 Hyperpolarization Induced by Parahydrogen in Reversible Exchange. *J. Phys. Chem. C* **121**, 6626-6634, doi:10.1021/acs.jpcc.6b12097 (2017).
